# Supplementary material for: A fungal avirulence factor encoded in a highly plastic genomic region triggers partial resistance to septoria tritici blotch
Source: New Phytol. 2018 Apr 25;219(3):1048–61. doi: 10.1111/nph.15180 (PMC6055703; doi:10.1111/nph.15180)
Supplement: Supplementary file 1 — Fig. S1 Manual annotation of putative effector genes in the QTL for virulence. Fig. S2 The gene QTL7_5 does not contribute to virulence. Fig. S3 Z09_7_00581 encodes the avirulence factor Avr3D1 and sequence modifications lead to evasion of recognition. Fig. S4 Avr3D1 does not explain all the differences in virulence between 3D1 and 3D7. Fig. S5 In vitro growth of the mutant lines was unaltered under several stress conditions. Fig. S6 Avr3D1 expression peaks at the end of the latent phase. Fig. S7 Specific recognition of Avr3D1 by certain wheat varieties leads to a reduction in pycnidia formation. Fig. S8 Synteny plot of the QTL between five Zymoseptoria tritici strains. Fig. S9 Frequency distribution of dN : dS ratios for all pairwise Avr3D1 haplotype comparisons. Fig. S10 Orthologous sequences of Avr3D1 identified in five strains of Zymoseptoria pseudotritici. Fig. S11 Orthologous sequences of Avr3D1 identified in four strains of Zymoseptoria ardabiliae. Table S1 Primers used in this study Table S3 Model test and parameter estimates of diversifying selection with Paml based on the total Avr3D1 data set Notes S1 Population genetic analysis. [file NPH-219-1048-s001.pdf]

***New Phytologist* Supporting Information**

Article title: A fungal avirulence factor encoded in a highly plastic genomic region triggers partial resistance to septoria tritici blotch

Authors: Lukas Meile, Daniel Croll, Patrick C. Brunner, Clémence Plissonneau, Fanny E. Hartmann, Bruce A. McDonald and Andrea Sánchez-Vallet

Article acceptance date: 20 March 2018

The following Supporting Information is available for this article:

Fig. S1. Manual annotation of putative effector genes in the QTL for virulence.

Fig. S2. The gene *QTL7_5* does not contribute to virulence.

Fig. S3. *Z09_7_00581* encodes the avirulence factor Avr3D1 and sequence modifications lead to evasion of recognition**.**

Fig. S4. *Avr3D1* does not explain all the differences in virulence between 3D1 and 3D7.

Fig. S5. *In vitro* growth of the mutant lines was unaltered under several stress conditions.

Fig. S6. *Avr3D1* expression peaks at the end of the latent phase.

Fig. S7. Specific recognition of Avr3D1 by certain wheat varieties leads to a reduction in pycnidia formation.

Fig. S8. Synteny plot of the QTL between five *Zymoseptoria tritici* strains.

Fig. S9. Frequency distribution of dN : dS ratios for all pairwise *Avr3D1* haplotype comparisons**.**

Fig. S10. Orthologous sequences of Avr3D1 identified in five strains of *Zymoseptoria pseudotritici*.

Fig. S11. Orthologous sequences of Avr3D1 identified in four strains of *Zymoseptoria ardabiliae*.

Table S1. Primers used in this study.

Table S2. Effector gene cluster annotation. gff file of the manually reannotated effector genes identified in the QTL. (See separate file).

Table S3. Model test and parameter estimates of diversifying selection with PAML based on the total *Avr3D1* data set.

Notes S1. Population genetic analysis

Fig. S1. Manual annotation of putative effector genes in the QTL for virulence

(a) RNA reads from the *Z. tritici* reference genome IPO323 mapped to gene *Z09_7_00581* (Rudd *et al.*, 2015). Possible start and stop codons are shown in green and red, respectively, for all forward reading frames. The previous model of *Z09_7_00581* (Grandaubert *et al.*, 2015, equivalent to JGI model) and the manually curated model are represented by blue boxes. (b) RNA reads of 3D7 genome (Palma-Guerrero *et al.*, 2016) mapped against the region containing the cluster of four genes predicted to encode SSPs.

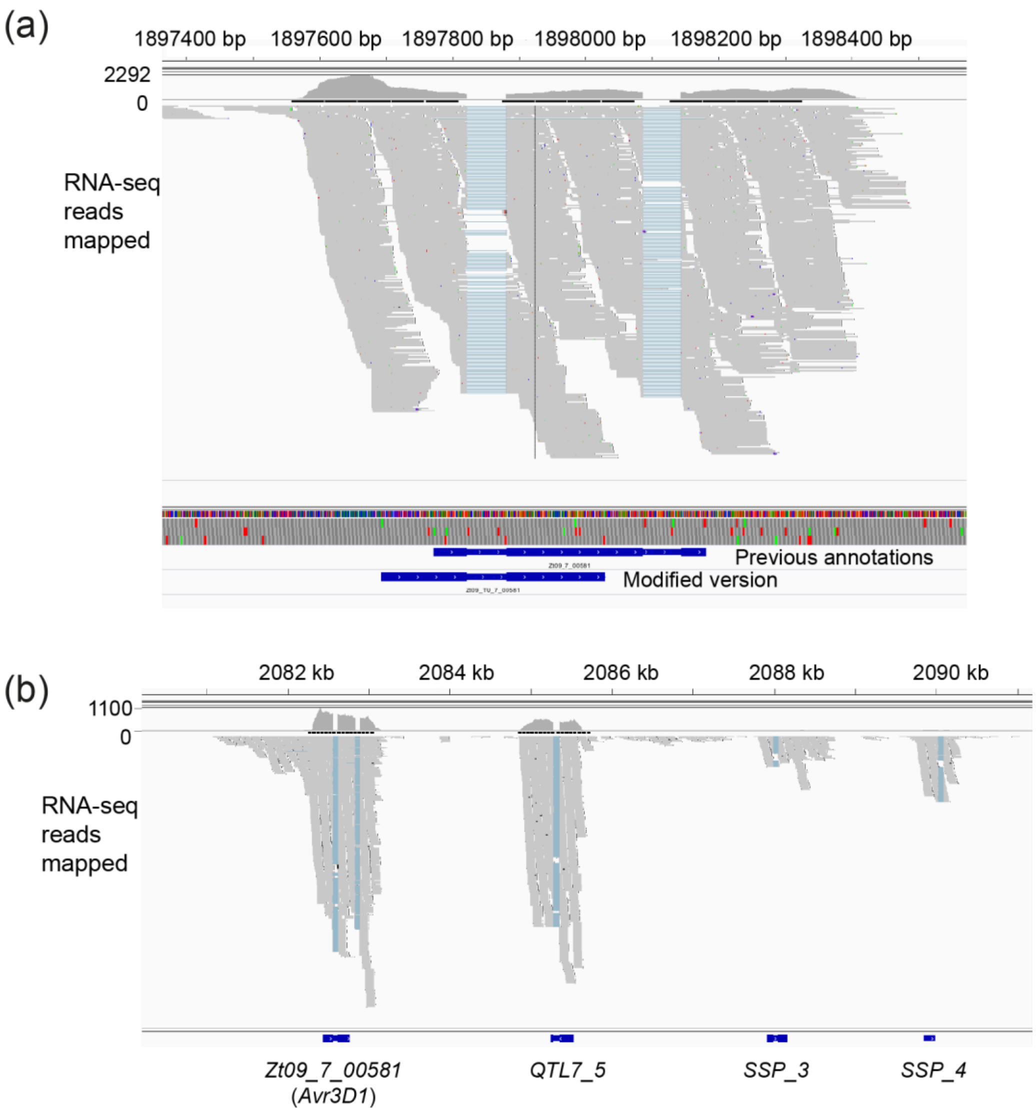

Fig. S2. The gene *QTL7_5* does not contribute to virulence

Percentage of leaf area covered by lesions (PLACL, upper panel) produced on the wheat cultivar Runal by the *Z. tritici* wild type (wt, red), three or four independent knockout (KO) lines of the gene *QTL7_5* (Δqtl7_5, green), two ectopic controls lines of the transformation (blue) and mock treatment (purple). (a) Mutants obtained in the 3D1 background. (b) Mutants in the 3D7 background. Red dots represent the median of at least seven leaves (panel A) or twelve leaves (panel B; except for the mock treatment, for which eight leaves were used), error bars represent 95% confidence intervals of the medians and black dots represent individual data points. dpi = days post inoculation. Note that plants used for panel A were regularly trimmed to the second true leaf. No statistical differences between knockout mutants and control lines was observed (α=0.01, Kolmogorov-Smirnov test).

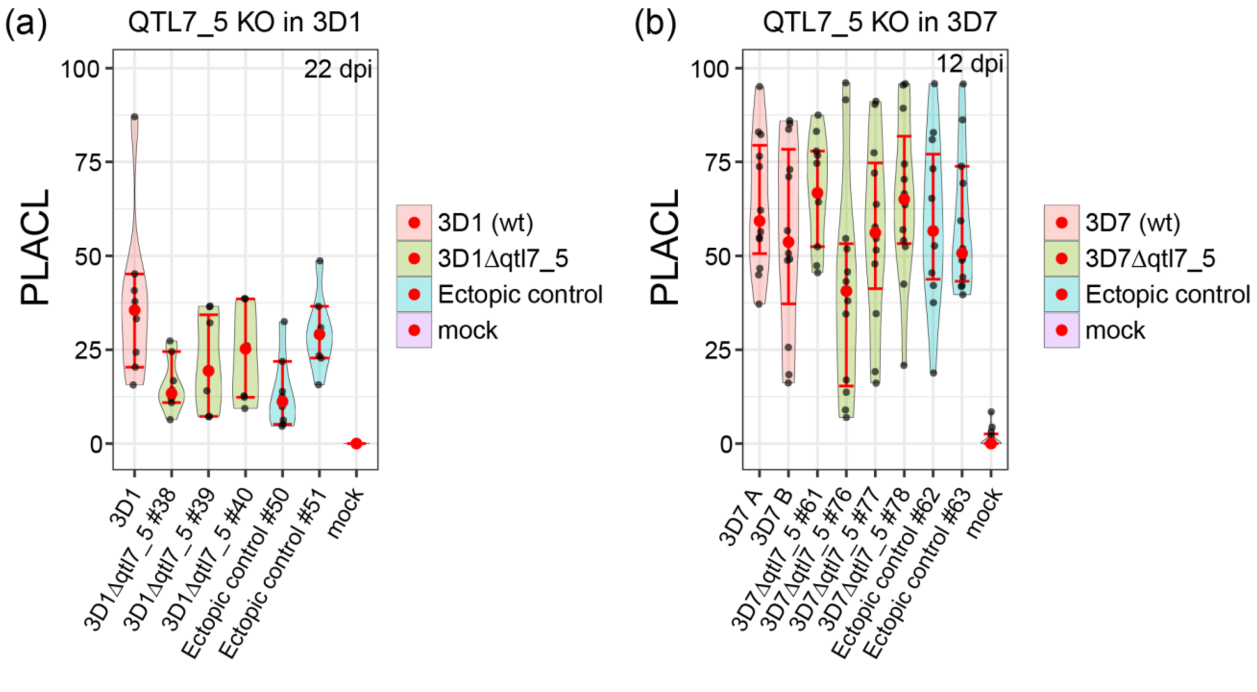

Fig. S3. *Z09_7_00581* encodes the avirulence factor Avr3D1 and sequence modifications lead to evasion of recognition

Percentage of leaf area covered by lesions (PLACL, upper panels) and pycnidia/cm² lesion (lower panels) produced on wheat cultivar Runal by the *Z. tritici* wild type (wt, red), the knockout (KO) in *Avr3D1* (Δavr3D1, yellow), three or two independent mutant lines expressing the 3D1 allele (*Avr3D1*$_{3D1}$, green) or the 3D7 allele (*Avr3D1*$_{3D7}$, blue), the transformation control (transformed with the empty pCGEN vector, purple) and the mock treatment (pink). Mutants were obtained in the 3D1 background (left) and in the 3D7 background (right). Two independent cultures of the wild type and the knockouts (A and B) were used for infection. Red dots represent the median of at least fourteen leaves (except for the mock treatment, for which at least eight leaves were used), error bars represent the 95% confidence intervals of the medians and black dots represent individual data points. Asterisks represent statistical differences between wild types (3D1 or 3D7 culture A, respectively, p-value < 0.01, Kolmogorov-Smirnov test) and mutants. dpi = days post inoculation. The experiments were repeated twice with similar results.

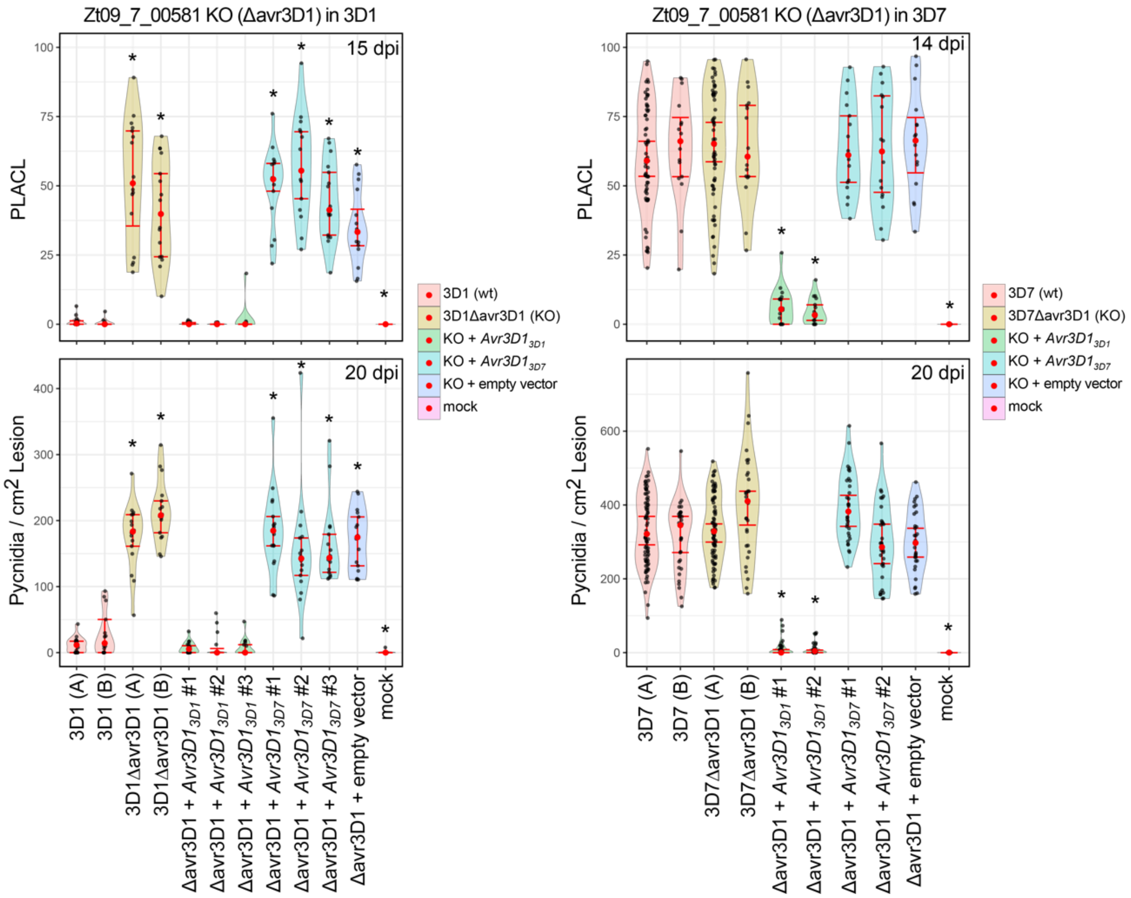

Fig. S4. *Avr3D1* does not explain all the differences in virulence between 3D1 and 3D7

Percentage of leaf area covered by lesions (PLACL) at 17 dpi (2nd true leaf, upper panel) and pycnidia density (pycnidia / cm^2 lesion) at 25 dpi (3rd true leaf, lower panel) produced on wheat cultivar Runal by *Z. tritici* 3D7, the wild type (wt) 3D1 (two independent inocula, A and B), four independent Avr3D1 knockout lines (KO, 3D1Δavr3D1) and two independent ectopic control lines of the transformation. Red dots represent the median of at least ten leaves (except for the mock treatment, for which at least four leaves were used), error bars represent 95% confidence intervals of the medians and black dots represent individual data points. dpi = days post inoculation. Asterisks indicate statistical differences between 3D1 wild type (A) and mutants (p-value < 0.01, Kolmogorov-Smirnov test).

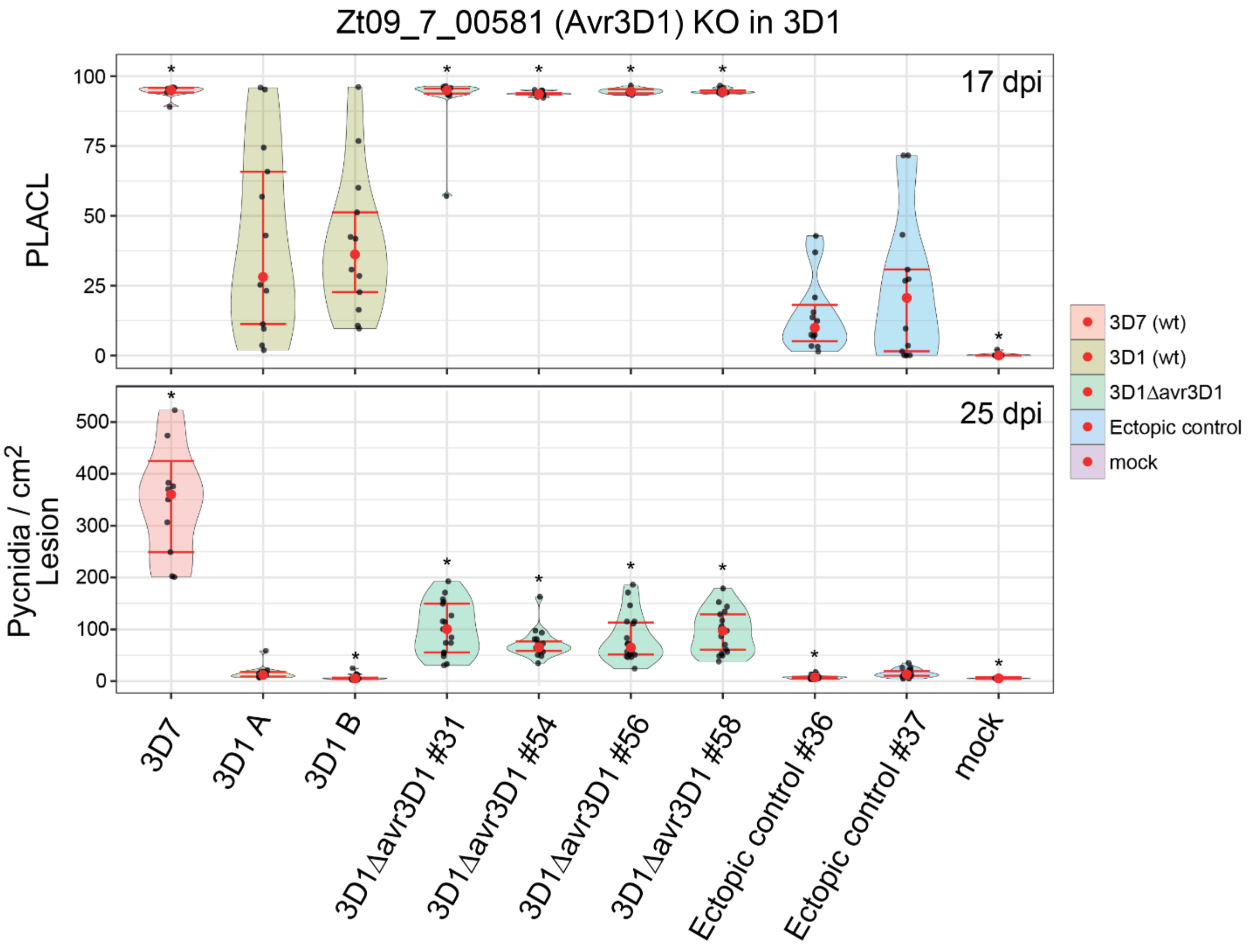

Fig. S5. *In vitro* growth of the mutant lines was unaltered under several stress conditions

Phenotypes of the *Z. tritici* wild types and the mutants in solid media. For each mutant and condition two drops of 2.5 µl at two different spore concentrations (10^6 and 10^5 spores/ml) are shown. All the plates were incubated at 18°C, unless indicated otherwise.

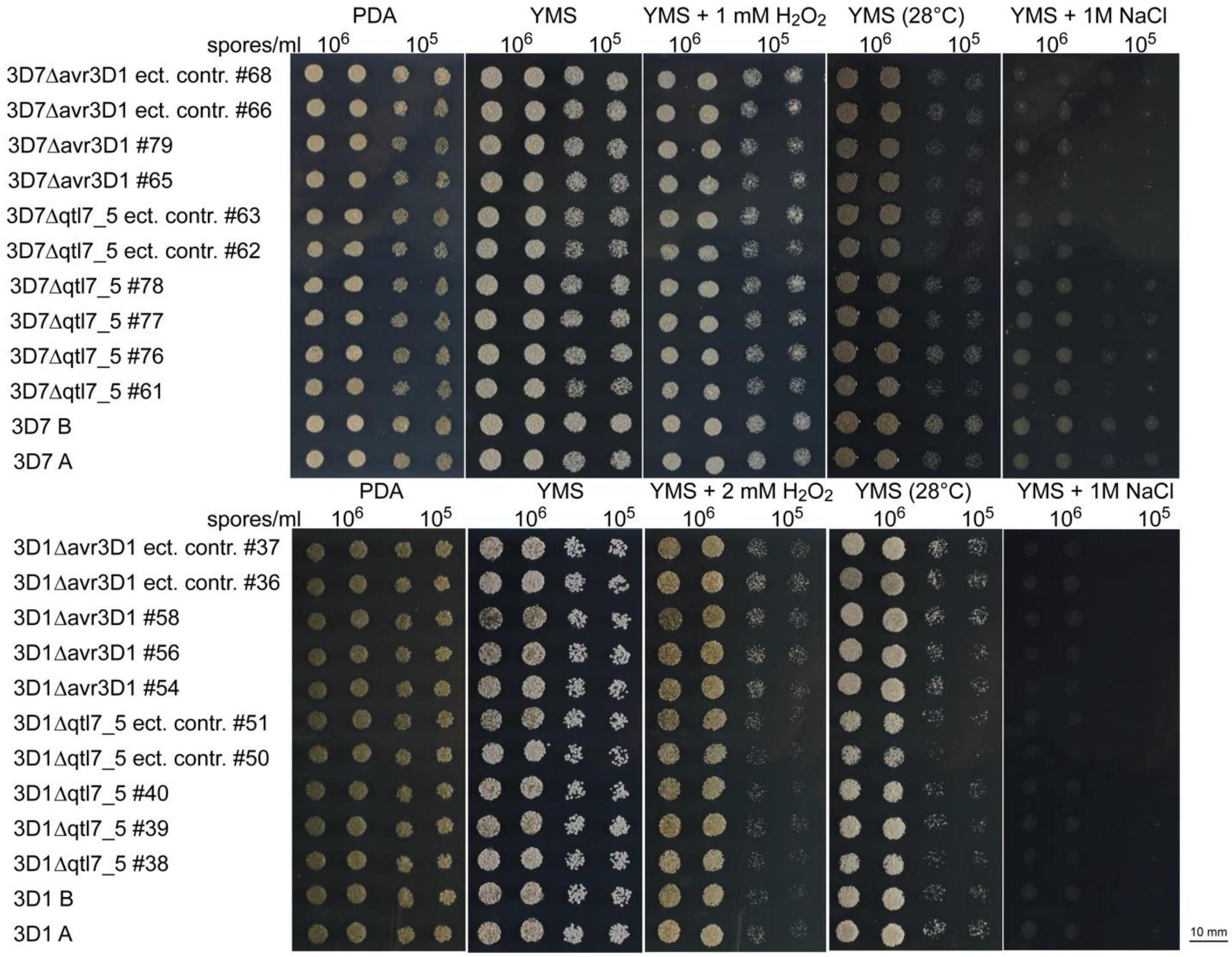

Fig. S6. *Avr3D1* expression peaks at the end of the latent phase

Expression profile of *Avr3D1* in *Z. tritici* 3D1 (red) and 3D7 (blue) during infection of the wheat cultivar Runal. The values shown are the average of three biological replicates of the relative expression levels of *Avr3D1* with respect to the reference *18S* rRNA gene. Error bars represent the 95% confidence intervals of the averages. Pictures show phenotypes of the infected leaves at each time point.

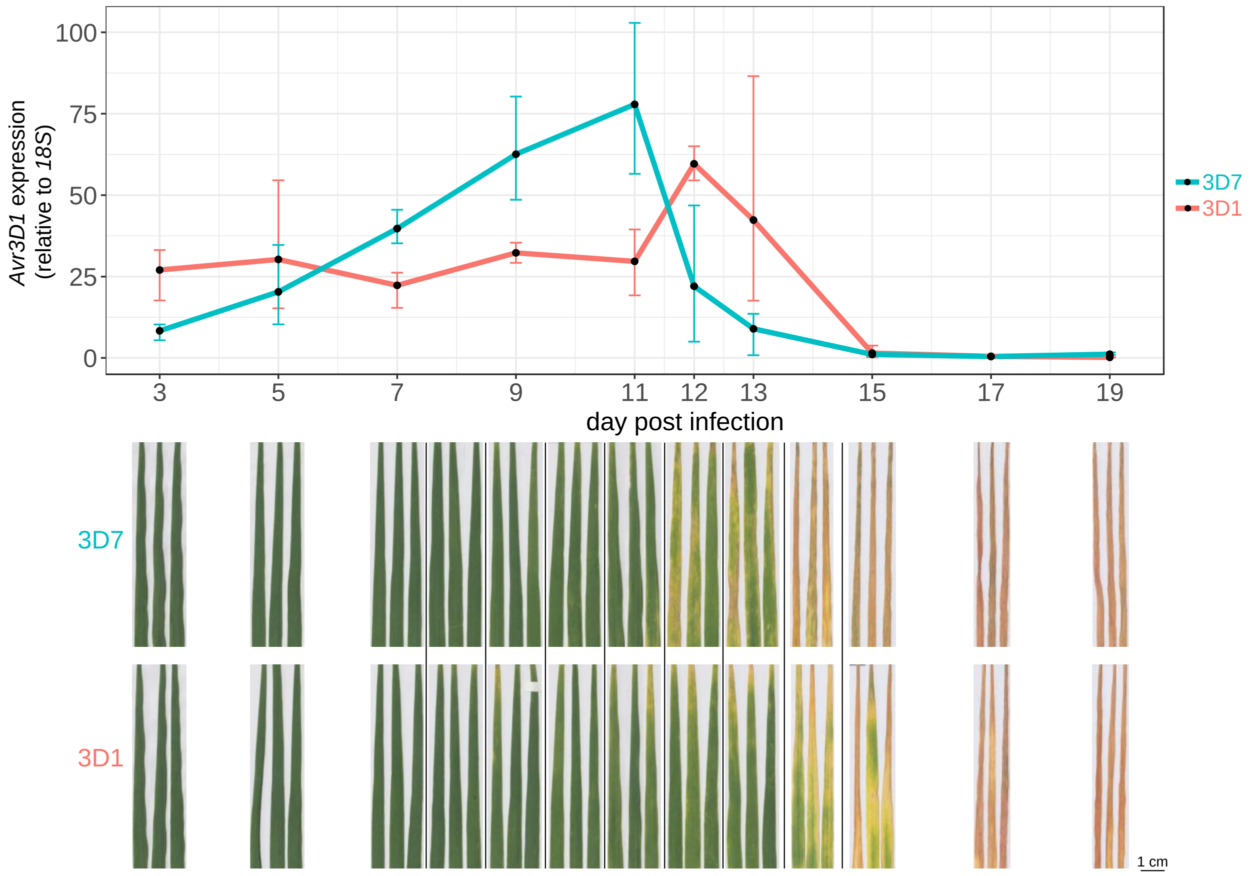

Fig. S7. Specific recognition of Avr3D1 by certain wheat varieties leads to a reduction in pycnidia formation

Violin plots showing pycnidia density (pycnidia/cm^2 lesion) produced by the *Z. tritici* wild type (wt) 3D1, the Avr3D1 knockout (3D1Δavr3D1) and the mock control in seventeen wheat cultivars. Harvesting time points varied because of cultivar-specific infection dynamics. Either second or third leaves were harvested depending on where more pycnidia were observed, except for the cultivar Runal and ST6 in which leaves from both positions were analyzed. Red dots represent the median of at least ten leaves (except for the mock treatment, for which at least four leaves were used), error bars represent 95% confidence intervals of the medians and black dots represent individual data points. Asterisks indicate statistical differences between wild type and knockout (p-value < 0.01, Kolmogorov-Smirnov test). Synthetic CS = Synthetic Chinese Spring; ST6= Estanzuela Federal; M6= M6 synthetic (W-7984), Kavkaz-K4500 = Kavkaz-K4500 L.6.A.4, dpi = days post inoculation. This experiment was repeated with cultivars Runal, Kavkaz-K4500 L.6.A.4, ST6, TE-9111, Arina, Titlis, M6 and Bulgaria 88 and similar results were obtained.

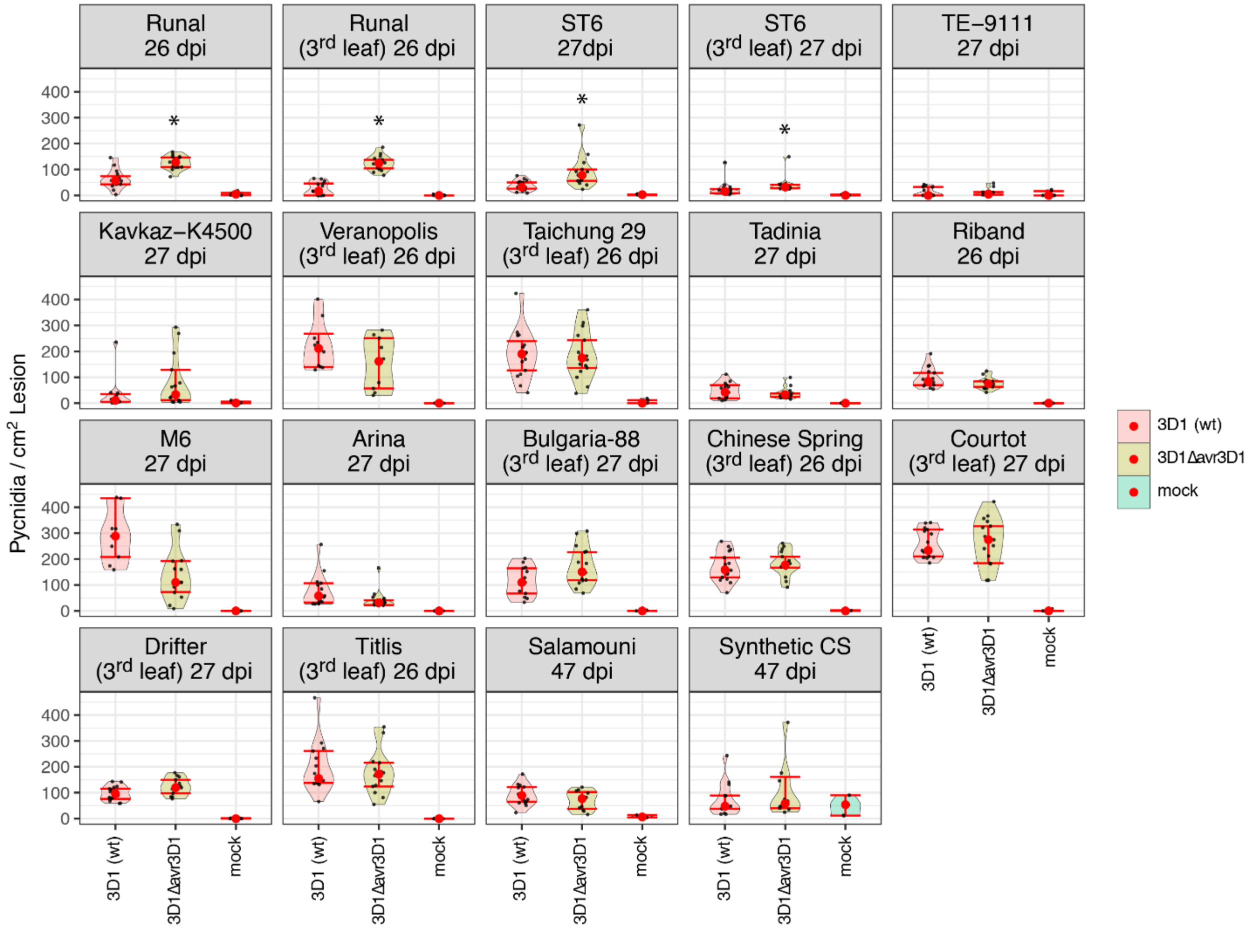

Fig. S8. Synteny plot of the QTL between five *Zymoseptoria tritici* strains

Synteny plot comparing the QTL region for virulence between strains IPO323, 3D7, 3D1, 1E4 and 1A5. The borders of the 95% confidence interval of the QTL in 3D7 are marked by black vertical lines. Genes are represented by red arrows and transposable elements are represented by blue blocks. Collinear sequences between the two strains are shown in different shades of brown according to their sequence identity. Gene annotation used for all the strains correspond to Grandaubert *et al*., (2015) for simplicity. The transposable elements were classified according to the three-letter code described in Wicker *et al*. (2016): The first letter indicates the class (R = RNA class and D = DNA class); the second letter indicates the order (L = LTR, I = Line, T= TIR, Y= Crypton, H = Helitron); and the third letter indicates the superfamily (C = *Copia*, G = *Gypsy*, I = *I*, H= *PIF-Harbinger*, M = *Mutator*, X = unknown).

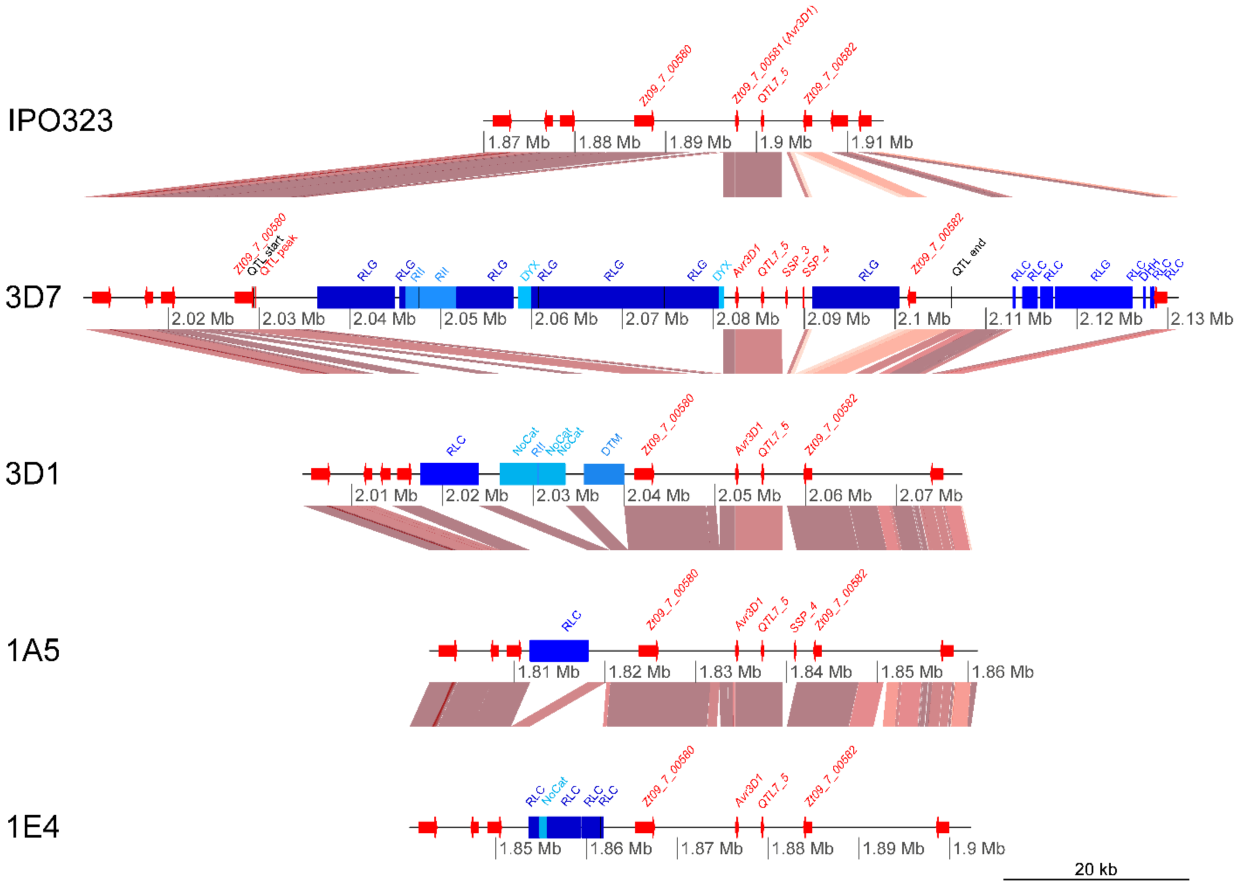

Fig. S9. Frequency distribution of dN : dS ratios for all pairwise *Avr3D1* haplotype comparisons. “inf” indicates infinite values due to comparisons among sequence pairs containing only nonsynonymous changes. Values above 1 indicate diversifying selection.

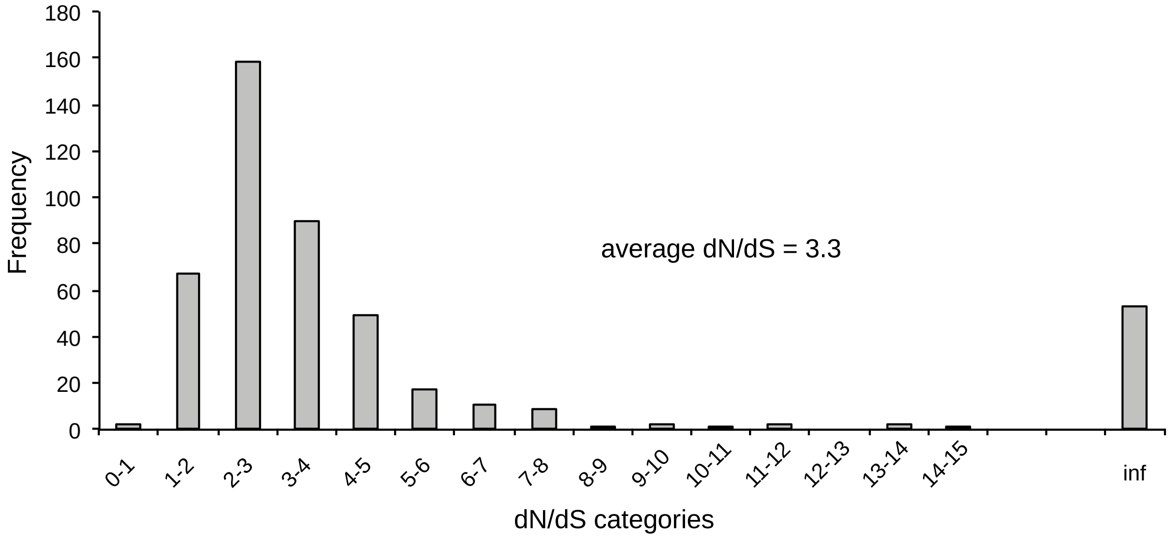

Fig. S10. Orthologous sequences of *Avr3D1* identified in five strains of *Zymoseptoria pseudotritici*. (a) Nucleotide variations of Iranian *Z. pseudotritici* strains STIR04 2.2.1 (AFIQ01), STIR04 3.11.1 (AFIO01), STIR04 4.3.1 (AFIR01), STIR04 5.3 (AFIS01), STIR04 5.9.1 (AFIT01) are shown with respect to Swiss *Z. tritici* reference strain 3D1. Dots indicate identical nucleotides, the grey region highlights the intron sequence. (b) Amino acid variations are shown with respect to Swiss *Z. tritici* reference strain 3D1. Dots indicate identical amino acids.

(a)

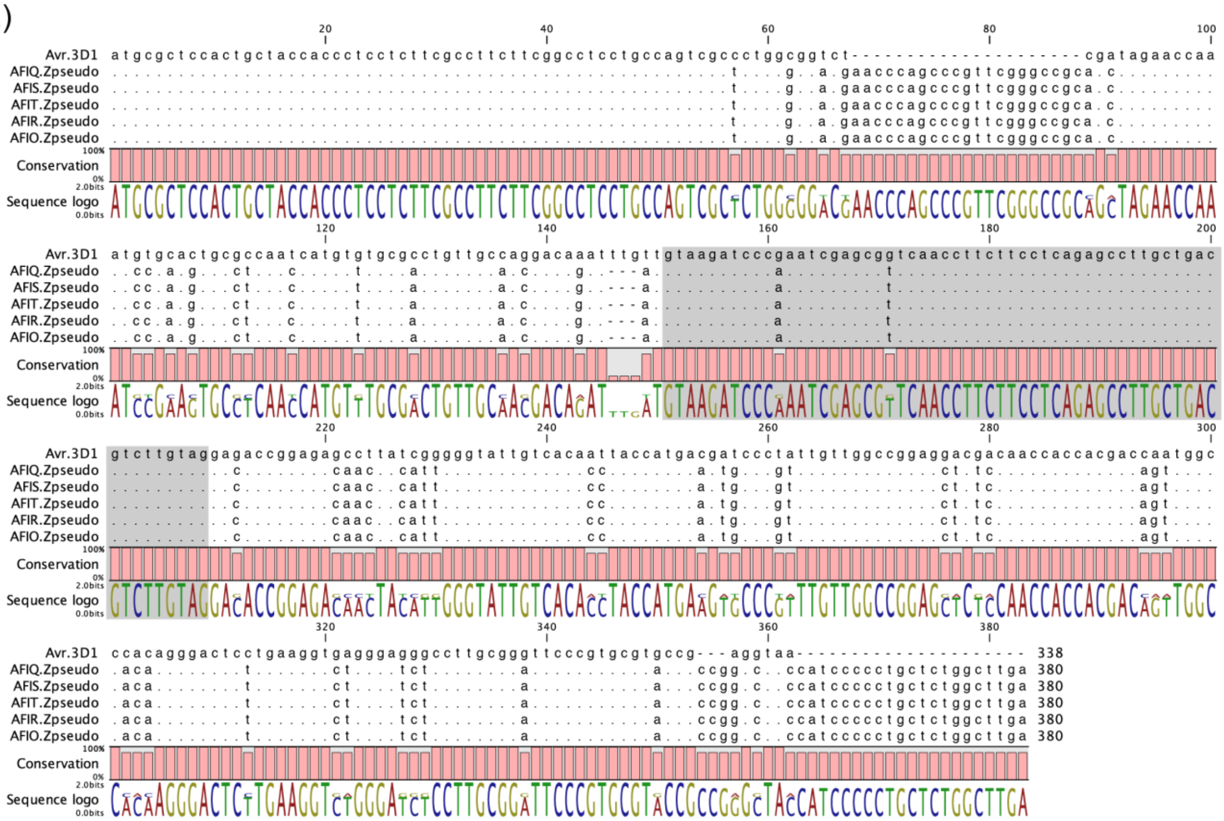

(b)

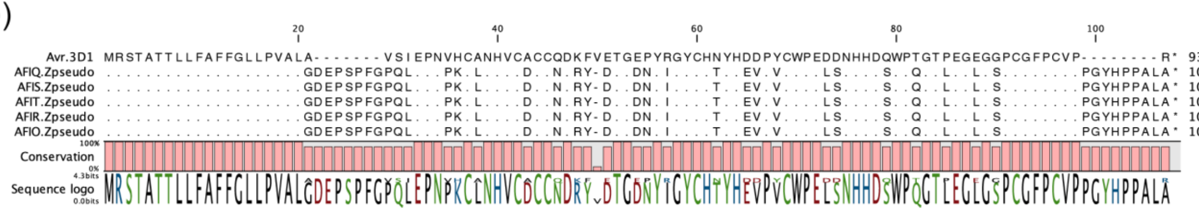

Fig. S11. Orthologous sequences of *Avr3D1* identified in four strains of *Zymoseptoria ardabiliae*. (a) Nucleotide variations of Iranian *Z. ardabiliae* strains STIR04 1.1.1 (AFIU01), STIR04 1.1.2 (AFIV01), STIR04 3.13.1 (AFIW01), STIR04 3.3.2 (AFIX01) are shown with respect to Swiss *Z. tritici* reference strain 3D1. Dots indicate identical nucleotides, grey region denotes the intron sequence. (b) Amino acid variations are shown with respect to the Swiss *Z. tritici* reference strain 3D1. Dots indicate identical amino acids.

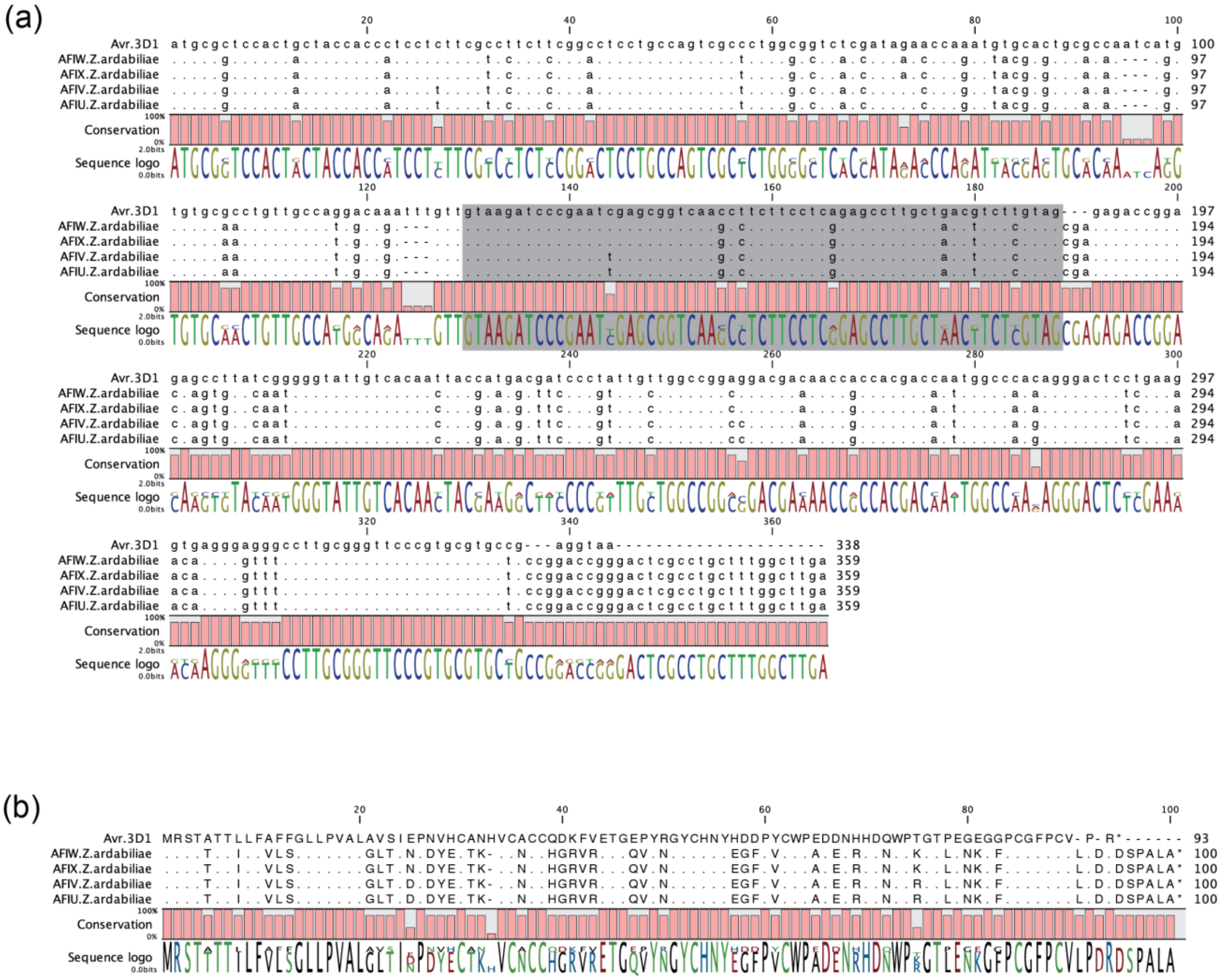

Table S1. Primers used in this study.

Number	Name	Sequence (5'-3')	Application
LMp_36	I-F_UF-00581KO_F	TAATTAAGATATCGAGCTCGGACT TCTTCCGACGACTTCC	Avr3D1 deletion (upstream flanking region; with LMp_37)
LMp_37	I-F_UF-00581KO_R	CTCCTTCAATATCAAAGCCGCATT GTGTCGAGGCTGGTG	Avr3D1 deletion (upstream flanking region; with LMp_36)
LMp_38	I-F_DF-00581KO_F	ATAGAGATCTGCTAGCCATCCTCC TCTTCGCCTTCTTCGG	Avr3D1 deletion (downstream flanking region; with LMp_39)
LMp_39	I-F_DF-00581KO_R	CAGTGCCAAGCTTGCATGCCAATA ATCCCATCCTACCTCGCC	Avr3D1 deletion (downstream flanking region; with LMp_38)
LMp_40	I-F_UF-3D7_5KO_F	CAGTGCCAAGCTTGCATGCCACT GCCAGATGTGTTCCTCAG	QTL7_5 deletion (upstream flanking region; with LMp_41)
LMp_41	I-F_UF-3D7_5KO_R	ATAGAGATCTGCTAGCCATCCATT GTTGTGGATGGGTTGC	QTL7_5 deletion (upstream flanking region; with LMp_40)
LMp_42	I-F_DF-3D7_5KO_F	CTCCTTCAATATCAAAGCCGGTTT CGCCATCTTCGCTGC	QTL7_5 deletion (downstream flanking region; with LMp_43)
LMp_43	I-F_DF-3D7_5KO_R	TAATTAAGATATCGAGCTCGGGCT TTCGTTCAGTCAACTCG	QTL7_5 deletion (downstream flanking region; with LMp_42)
LMp_26	Hyg_univ_F	CGGCTTTGATATTGAAGGAGC	Gene deletion (Hygromycin resistance gene; with LMp_27)
LMp_27	Hyg_univ_R	GATGGCTAGCAGATCTCTATTCC	Gene deletion (Hygromycin resistance gene; with LMp_26)
LMp_95	IF-581ect_F2	GCCGAATTCGAGCTCGCTACCTT GAGTGGACATGAGGA	Ectopic integration of Avr3D1$_{3D1}$ (with LMp_96)
LMp_96	IF-581ect_R2	CATGGTGGAGTGAGGGGTACCAA TAATCCCATCCTACCTCGC	Ectopic integration of Avr3D1$_{3D1}$ (with LMp_95)
LMp_97	IF-XhoI-581prom_F	TCGCCGTGCCTGGGCT	Ectopic integration of Avr3D1$_{3D7}$ (with LMp_98)
LMp_98	IF-581prom_R	GCTGGTGTCGTGGTGTTGTG	Ectopic integration of Avr3D1$_{3D7}$ (with LMp_97)
LMp_99	IF-581CDS_F	CACAACACCACGACACCAGC	Ectopic integration of Avr3D1$_{3D7}$ (with LMp_100)
LMp_100	IF-XhoI-581CDS_R	TGGACTCCTTCTCGCTCTCG	Ectopic integration of Avr3D1$_{3D7}$ (with LMp_99)
ASVp_1	100846.UP_F	CACTCTCGGAGCACTCCTCGATT	Verification of Δ581 lines (with LMp_4)
LMp_4	d3_100846	AGTCAATGGACCTGGCTCAAC	Verification of Δ581 lines (with ASVp_1)
LMp_16	3D7_5KO_scr_R	CCCAAAGAATCAAAGGTGTG	Verification of ΔQTL7_5 lines (with LMp_19 or LMp_76)
LMp_19	u1_3D7_5	GCACCATTGAACGTCCTGAG	Verification of Δ QTL7_5 lines (with LMp_16)
LMp_66	581_wt_F	ATGCGCTCCACTGCTACCAC	Verification of Δ581 lines (with ASVp_2)
ASVp_2	100846.Down_R	AGCGCACTTGAATACGACTACATG	Verification of Δ581 lines (LMp_66)
LMp_76	3D7_5_wt_F	TCCACAACAATGCGTTTCG	Verification of Δ QTL7_5 lines (with LMp_16)
LMp_77	581_q_F	GAAGGAGTCCATTGCGTTTC	qRT-PCR (with LMp_78)
LMp_78	581_q_R	TGAAACATCCTTCTTCCCACG	qRT-PCR (with LMp_77)
ASVp_3	Zt18S_F	CCAGCAAATCCTTCGATCTC	qRT-PCR reference gene (with Zt18S_R)
ASVp_4	Zt18S_R	CCACTTTGACATTTCCACACC	qRT-PCR reference gene (with Zt18S_F)
ASVp_5	FL_TFIIIC1_F	TGCTCAGATTGTGCGAAGAC	qPCR reference gene (with FL_TFIIIC1_R)
ASVp_6	FL_TFIIIC1_R	TCGTAGTCCGATACCATGAGG	qPCR reference gene (with FL_TFIIIC1_F)
ASVp_7	GenR_q_F	CTGTGCTCGACGTTGTCACT	qPCR for copy number detection (with GenR_q_R)
ASVp_8	GenR_q_R	ATACTTTCTCGGCAGGAGCA	qPCR for copy number detection (with GenR_q_F)
ASVp_9	HygR_q_F	CGTCTGCTGCTCCATACAAG	qPCR for copy number detection (with HygR_q_R)
ASVp_10	HygR_q_R	CTCGATGAGCTGATGCTTTG	qPCR for copy number detection (with HygR_q_F)

Table S2. Effector gene cluster annotation. gff file of the manually reannotated effector genes identified in the QTL. (See separate file).

Table S3. Model test and parameter estimates of diversifying selection with PAML based on the total *Avr3D1* data set.

Model	parameter estimates			L	PSS
M0: one ratio	ω = 4.24			-1385	none
M1: neutral	p0 = 0.57<br>ω0 = 0	p1 = 0.43<br>ω1 = 1		-1350	na
M2: selection	p0 = 0.43<br>ω0 = 0	p1 = 0.30<br>ω1 = 1	p2 = 0.27<br>ω2 = 10.9	-1295	18
M7: beta	p = 0.01	q = 0.01		-1361	na
M8: beta & ω	p = 0.01<br>p0 = 0.74	q = 0.01<br>p1 = 0.26	ω = 10.8	-1295	20

p0, p1, p2 = proportion of codon sites having the ω ratios of the respective site class

p, q = parameters of the beta distribution for ω

L = likelihood estimate

PSS = number of positively selected sites with probability $p > 0.99$

ω, omega = dN/dS

na = not applicable

Notes S1. Population genetic analysis. Strong evidence for non-neutral evolution in *Avr3D1*

Thirty-one different alleles were identified for the gene *Avr3D1* in *Z. tritici*. There were 65 polymorphic sites with a total of 78 substitutions. The distribution of dN/dS estimates averaged over the entire gene was calculated (Fig. S9) for all pairwise sequence comparisons. Fifty-three comparisons resulted in “infinite” values of dN/dS, indicating that only nonsynonymous changes were detected between those sequence pairs. Excluding these values, the average dN/dS ratio over all codon haplotypes was 3.3, which is significantly larger than the ratio of 1.0 expected for a neutral gene.

We also conducted codon-based selection analyses using PAML (Table S3). The maximum likelihood approach indicated that evolutionary models with variable dN/dS among codon sites (models M7 and M8) fitted the data significantly better than the one ratio model M0 ($p < 0.001$ for both comparisons). Furthermore, the neutral model M7 (ln -1361) fitted the data less well than the selection model M8 (ln -1295, $p < 0.001$), suggesting that diversifying selection is acting on the *Avr3D1* gene. Out of 96 codon sites, 58 were estimated to be under purifying selection, 3 were neutral, and 35 were in the category of diversifying selection, with an estimated dN/dS of 10.79, suggesting that strong diversifying selection is operating at these sites. These inferred selection categories for each site are color-coded in Fig. 4b.

References

Grandaubert J, Bhattacharyya A, Stukenbrock EH. 2015. RNA-seq-based gene annotation and comparative genomics of four fungal grass pathogens in the genus *Zymoseptoria* identify novel orphan genes and species-specific invasions of transposable elements. *G3 : genes - genomes - genetics* **5**(7): 1323-1333.

Palma-Guerrero J, Torriani SFF, Zala M, Carter D, Courbot M, Rudd JJ, McDonald BA, Croll D. 2016. Comparative transcriptomic analyses of *Zymoseptoria tritici* strains show complex lifestyle transitions and intraspecific variability in transcription profiles. *Molecular Plant Pathology* **17**(6): 845-859.

Rudd JJ, Kanyuka K, Hassani-Pak K, Derbyshire M, Andongabo A, Devonshire J, Lysenko A, Saqi M, Desai NM, Powers SJ, et al. 2015. Transcriptome and metabolite profiling of the infection cycle of *Zymoseptoria tritici* on wheat reveals a biphasic interaction with plant immunity involving differential pathogen chromosomal contributions and a variation on the hemibiotrophic lifestyle definition. *Plant Physiology* **167**(3): 1158-1185.
